# Sacrocolpopexy with Polypropylene Tape as Valuable Surgical Modification during Cystectomy with Orthotopic Ileal Bladder: Functional Results

**DOI:** 10.1155/2015/306191

**Published:** 2015-02-19

**Authors:** Marcin Życzkowski, Bartosz Muskała, Zbigniew Kaletka, Piotr Bryniarski, Krzysztof Nowakowski, Rafał Bogacki, Andrzej Paradysz

**Affiliations:** Department and Clinic of Urology, Medical University of Silesia, 41-800 Katowice, Poland

## Abstract

*Introduction*. Urinary diversion is very often associated with urinary retention and urinary incontinence. In this study, a surgical modification during cystectomy with orthotopic ileal neobladder is presented. *Material and Methods*. Female patients enrolled in the study (*n*-24) were subjected to sacrocolpopexy during the operation. Apart from oncological control, the follow-up consisted of 1-hour inlay test and questionnaires (UDI-6 and IIQ-7) in the 3rd, 6th, and 12th month after the operation. In the 12th month after the surgery, the urodynamic pressure-flow test was performed. Outcomes were compared with the control group (*n*-18) in which sacrocolpopexy was not implemented. *Results*. The study group was characterised by reduced urinary retention and improved continence. *Conclusion*. Sacrocolpopexy during cystectomy with orthotopic ileal bladder is a valuable surgical method which provides patients with a better quality of life.

## 1. Introduction

Bladder cancer is the 9th most frequently occurring cancer among all malignancies. It is found 3-4 times more often in men than in women [1]. About 30% of newly diagnosed bladder cancers are muscle-invasive where the method of choice for treatment is a radical cystectomy [2]. There are many methods of urinary diversion after cystectomy but creating a neobladder is the one which best corresponds to natural anatomical conditions. Unfortunately, in a significant percentage of patients, urinary retention occurs which requires catheterization of the intestinal reservoir. On the other hand, achieving the urinary continence grade being satisfactory for doctors and above all for women who underwent this kind of operation is not always possible. That is why, in order to avoid these complications, there are still attempts to modify already well-tried operation methods or just to introduce new ones. The surgery for pelvic organ prolapse focuses on the restoration of the vaginal anatomy and normal bladder and bowel function [3]. It is proven that this method is superior to other surgical techniques in terms of restoration of the normal vaginal axis and maintenance of vaginal capacity in cases of pelvic organ prolapse [4]. The reinforcement of the pelvic floor seems to be also useful after cystectomy with neobladder creation.

## 2. Objectives

The aim of this open-label study is the effectiveness assessment of modification of the operation technique during radical cystectomy in the form of sacrocolpopexy with a polypropylene tape.

## 3. Material and Methods

**Figure 1:**
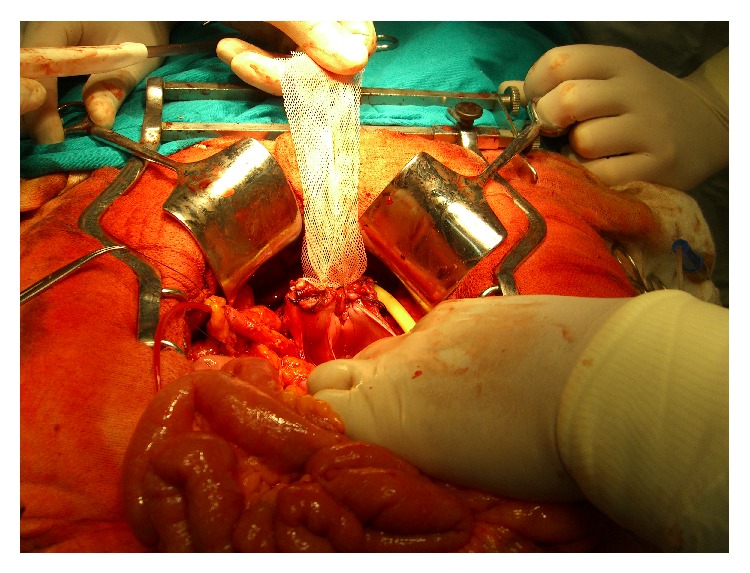
Polypropylene mesh sutured to the vaginal stump.

42 female patients aged 54–66 (average 59,7) with muscle-invasive bladder cancer and qualified for surgical treatment were enrolled in the research. 24 of them were in the study group. The control group consisted of 18 patients (Table 1). A simple randomization was used to divide assigned patients to the two above mentioned groups. All females underwent radical cystectomy. The urinary bladder, uterus with appendages, and anterior vaginal wall were excised. In any case the urinary diversion with Studer ileal bladder was performed. 45 cm long segment of ileum which was 25 cm proximal to the ileocecal valve was used. Ureters were implanted in the proximal part of pouch by a standard end-to-side method using “double J” ureteral stents. Before the anastomosis of enteral pouch with urethra was performed, each patient from the study group underwent sacrocolpopexy with a 8 × 3 cm tape made from a polypropylene mesh Dallop PP TDM KTM designed for the surgical treatment of hernias. A vaginal stump was sutured to promontorium with the mesh making C-shape suture line (Figure 1). The anastomosis of enteral pouch with urethra was performed with 6–10 single sutures.

The females were taken under accurate observation which lasted from 12 to 48 months. The oncological control was routine: chest, abdomen, and pelvis imaging (computed tomography) every 3 to 6 months for 2 years based on the risk of recurrence. Particular attention was paid to functional outcomes of urinary drainage with an ileal neobladder, especially to evaluate urinary retention grade and urinary incontinence. A follow-up planned for the first 12 months after the operation proceeded as follows: a one-hour inlay test, UDI-6 (Urinary Distress Inventory) and IIQ-7 (Incontinence Impact Questionnaire) questionnaires were performed in the 3rd, 6th, and 12th month after the operation. The inlay test was performed as follows: patients were asked to empty their bladders and to drink 500 mL of water in 15 minutes. After a 30-minute rest they performed similar activities: climbing stairs, walking, and coughing. The test was defined as positive if the urine loss was greater than 2 grams (pad weighed before and after the test). Urodynamic study in the form of pressure-flow study by means of Medtronic Duet G2 device was performed in the 12th month.

All data was checked for normality with Kolmogorov-Smirnov test. For analysis of continuous variables without normal distribution nonparametric Mann-Whitney* U* test was used. For analysis of categorical variables chi-square was used. Statistical examination was conducted with the aid of Statistica Statsoft v 9.0. *P* values <5% were considered statistically significant.

## 4. Results

Serious postoperative complications were not observed among patients from the examined group. In 4 patients there was prolonged urinary leakage and in 4 female patients postoperative ileus lasting over 5 days occurred. None of the females needed reoperation due to complications. With regard to lymph node metastases, the adjuvant chemotherapy was applied in 6 female patients. The average result of the UDI-6 questionnaire in the 3rd, 6th, and 12th month was, respectively, 2,0, 1,5, and 0,83. The average result of IIQ-7 questionnaire in the 3rd, 6th, and 12th month was, respectively, 1,5, 0,9, and 0,57. A one-hour inlay test in the 3rd, 6th, and 12th month of observation was negative in, respectively, 75% (*n*-18), 87,5% (*n*-21), and 91,6% (*n*-22) of patients. Urodynamic study was performed in the 12th month of observation in 20 female patients. The average cystometric capacity of a neobladder was 471 mL (350–576). All created neobladders were characterized by low intravesical pressure (mean 17,4 cm H_2_O). Slight urinary leakage while coughing was observed in 10% (*n*-2) of females with abdominal leak point pressure (ALPP) > 90 cm H_2_O). Only one of the operated female patients needed clean intermittent catheterization (CIC) due to the large amount of urinary retention after the micturition (400 mL). In the rest of 23 patients, the capacity of urinary retention after the micturition was about 65 mL (Tables 2, 3, and 4).

The results in the control group were as follows: the average result of the UDI-6 questionnaire in the 3rd, 6th, and 12th month was 2,9, 2,5, and 1,4, respectively. The average result of IIQ-7 questionnaire in the 3rd, 6th, and 12th month was, respectively, 2,5, 2,0, and 1,9. The inlay test in the 3rd, 6th, and 12th month was negative in 44% (*n*-8), 61% (*n*-11), and 67% (*n*-12) of female patients. In the urodynamic study performed in the 12th month of observation, the average cystometric capacity of a neobladder was 450 mL. Mean intravesical pressure by maximal cystometric capacity was 18 cm H_2_O. Positive coughing test occurred in 60% (*n*-12) of female patients with ALPP > 90 cm H_2_O. 10 patients (41,6%) needed CIC. The average urinary retention after the micturition was 184 mL.

Statistical significance was observed in the following values:

- a 1-hour inlay test questionnaire in the 3rd (*P*-0,03), 6th (*P*-0,02), and 12th month (0,003),
- UDI-6 in the 3rd, 6th, and 12th month (*P*-0,002),
- IIQ-7 in the 3rd, 6th, and 12th month (*P*-0,002),
- incidence of positive ALPP > 90 cm H_2_O in pressure-flow study (*P*-0,003),
- postvoid residual volume (*P*-0,04).

## 5. Discussion

The bladder cancer is the second most frequently occurring urinary system neoplasm. The cancer deriving from urothelium is the most common histological subtype constituting 90% of cases. The gold standard of treatment of the muscle-invasive bladder cancer is a radical cystectomy.

The proper selection of the method of urinary diversion in patients after cystectomy is one of the biggest challenges for urologists to deal with. Besides oncological radicality, the essence of the operation is to provide the patients with as good quality of life as possible. There are many kinds of neobladders using various segments of the digestive tract: from the small intestine to the sigmoid. None of them has significant advantage over the others with regard to providing patients the best quality of life [5].

The essence of a good functional result of a neobladder is effective bladder emptying and retained continence. According to some authors, sparing of sympathetic innervation during the cystectomy may be crucial to preserve urinary continence [6, 7]. Other authors claim that preserving the continence is still possible even if the autonomic innervation was not spared. In this case, the key to a good functional result is a delicate dissection of rhabdosphincter surroundings and preserving the pudendal innervation [8].

The Studer neobladder is an enteral pouch with good functional results [9]. It is said that patients with urinary diversion carried out by this method are characterized by total urinary continence in daytime in about 90% of cases [10]. Nocturnal incontinence takes place more often as it is about 60% [11]. A gradual improvement of continence proceeds from 6 to 12 months after creating a neobladder. Evaluation and potential therapy of urinary incontinence should be performed after the period in which the neobladder reaches its effective capacity.

The crucial problem concerning the effectiveness of a neobladder is urinary retention which constitutes the reason for recurrent urinary tract infections and urinary calculi formation. It is estimated that the necessity of clean intermittent catheterization (CIC) in patients with a neobladder made from the small intestine occurs in 20–70% of cases [12, 13] In most cases, the reason for ineffective emptying of neobladder in women is its anatomical factors in the form of neobladder folding and its displacement towards the back of pelvis. The reduction of the percentage of patients with urinary retention can be achieved by various modifications in the course of operation.

It is reported that the shorter segment of the small intestine (<40 cm), used in order to create a neobladder, results in the lower percentage of patients who need CIC [14]. The same effect may be observed in the patients who underwent sacrocolpopexy with or without a polypropylene tape. The short-term success rates of this method are reaching 90% in case of pelvic organ prolapse [4]. The abdominal sacral colpopexy comprises interposition of a synthetic mesh between the vagina and sacrum. This technique allows for more support of the vagina and distribution of tension over a larger surface area [15]. The attachment of vaginal stump to promontorium preserves the adequate axis of the neobladder.

The omental flap is also an effective surgical manoeuvre which ensures neobladder firmness. Comparing results in study and control group, this research proves that sacrocolpopexy during cystectomy significantly improves functional outcomes of urinary diversion in the form of less frequent and severe urinary retention and better diurnal continence.

## 6. Conclusions

Conclusions are drawn as follows.

- Fixation of vagina stump to promontorium is a simple method leading to stabilisation and reinforcement of the new-formed bladder neck.
- Sacrocolpopexy in patients after cystectomy with orthotopic ileal bladder is a valuable operative modification which clearly ameliorates functioning of the neobladder: reduces the postvoid residual volume and decreases necessity of CIC.
- It is possible that this mechanism, besides gentle preparing of urethral sphincter surroundings, significantly leads to continence improvement.
- Presented method requires further observation and investigation based on greater number of cases.

## Figures and Tables

**Table 1:** Preoperative patient's characteristics.

	Study group	Control group	*P*
Age (years)	59,5	57,7	0,3
T, *n* (%)			
2a	7 (29)	5 (27)	0,4
2b	3 (12,5)	3 (16,5)	
3a	10 (42)	7 (40)	
3b	4 (16,5)	3 (16,5)	
Grade, *n* (%)			
LG	4 (16)	3 (16)	0,3
HG	20 (84)	15 (84)	
BMI (kg/cm²)	21,3	22,1	0,3

T: tumour staging, LG: low grade, HG: high grade, and BMI: Body Mass Index.

**Table 2:** Postoperative 1-hour inlay test results.

	Study group	Control group	*P*
1-hour inlay test at 1st month, *n* (%)			
Negative	18 (75)	8 (44,4)	0,03
Positive	6 (25)	10 (55,6)	
1-hour inlay test at 6thmonth, *n* (%)			
Negative	21 (87,5)	11 (61)	0,02
Positive	3 (12,5)	7 (39)	
1-hour inlay test at 12th month, *n* (%)			
Negative	22 (91,7)	12 (67)	0,003
Positive	2 (8,3)	6 (33)	

**Table 3:** Quality of life parameters after surgery (UDI-6 and IIQ-7 questionnaires results).

	Study group	Control group	*P*
UDI-6 score (avg.) at months			
3	2,0 ± 0,29	2,9 ± 0,25	0,02
6	1,5 ± 0,1	2,5 ± 0,2	
12	0,8 ± 0,05	1,4 ± 0,1	
IIQ-7 score (avg.) at months			
3	1,5 ± 0,2	2,5 ± 0,21	0,002
6	0,9 ± 0,11	2,0 ± 0,12	
12	0,57 ± 0,1	1,8 ± 0,15	

UDI-6: Urogenital Distress Inventory and IIQ-7: Incontinence Impact Questionnaire.

**Table 4:** Urodynamic parameters after surgery (pressure-flow study).

	Study group	Control group	*P*
Average cystometric capacity (mL)	471 ± 20,3	452 ± 23,1	0,3
Average pressure at max capacity (cmH_2_O)	17,4 ± 3,6	18 ± 3,1	0,3
ALPP >90 cm H_2_O positive, *n* (%)	2 (8,3)	12 (66,7)	0,03
Average PVR (mL)	65 ± 10,3	184 ± 15,1	0,04

ALPP: abdominal leak point pressure and PVR: postvoid residual volume.
